# Supplementary material for: Hepatocyte growth factor activator inhibitor-2 rapidly inactivates airway-expressed human type II transmembrane serine proteases
Source: J Biol Chem. 2026 Aug 12;302(10):113440. doi: 10.1016/j.jbc.2026.113440 (PMC13578396; doi:10.1016/j.jbc.2026.113440)
Supplement: Supplementary file 1 [file mmc1.pdf]

Supporting Information

for

Hepatocyte growth factor activator inhibitor-2 rapidly inactivates airway-expressed human Type II Transmembrane Serine Proteases

Bryan J. Fraser[1,2]*, Ryan P. Wilson[1], Jackie Lac[1], Olzhas Ilyassov[1], Almagul Seitova[1,2], Yanjun Li[3], Ashley Hutchinson[1,2], Yen-Yen Li[1], Peter Loppnau[1,2], Maria Kutera[4], Gregg B. Morin[5,6,7]*, Francois Benard[6,8]*, and Cheryl H. Arrowsmith[1,2,4]*.

*Corresponding authors

Author Affiliations

1-Structural Genomics Consortium Toronto, Toronto, Ontario Canada
2-Princess Margaret Cancer Centre, University Health Network, Toronto, Ontario Canada
3-Department of Medicine, University of Toronto, Toronto, Ontario Canada
4-Department of Medical Biophysics, University of Toronto, Toronto, Ontario Canada
5-Canada's Michael Smith Genome Sciences Centre, Vancouver, British Columbia Canada
6-British Columbia Cancer Research Institute, Vancouver, British Columbia Canada
7-Department of Medical Genetics, University of British Columbia, Vancouver, British Columbia Canada
8-Department of Radiology, University of British Columbia, Vancouver, British Columbia Canada

Figures

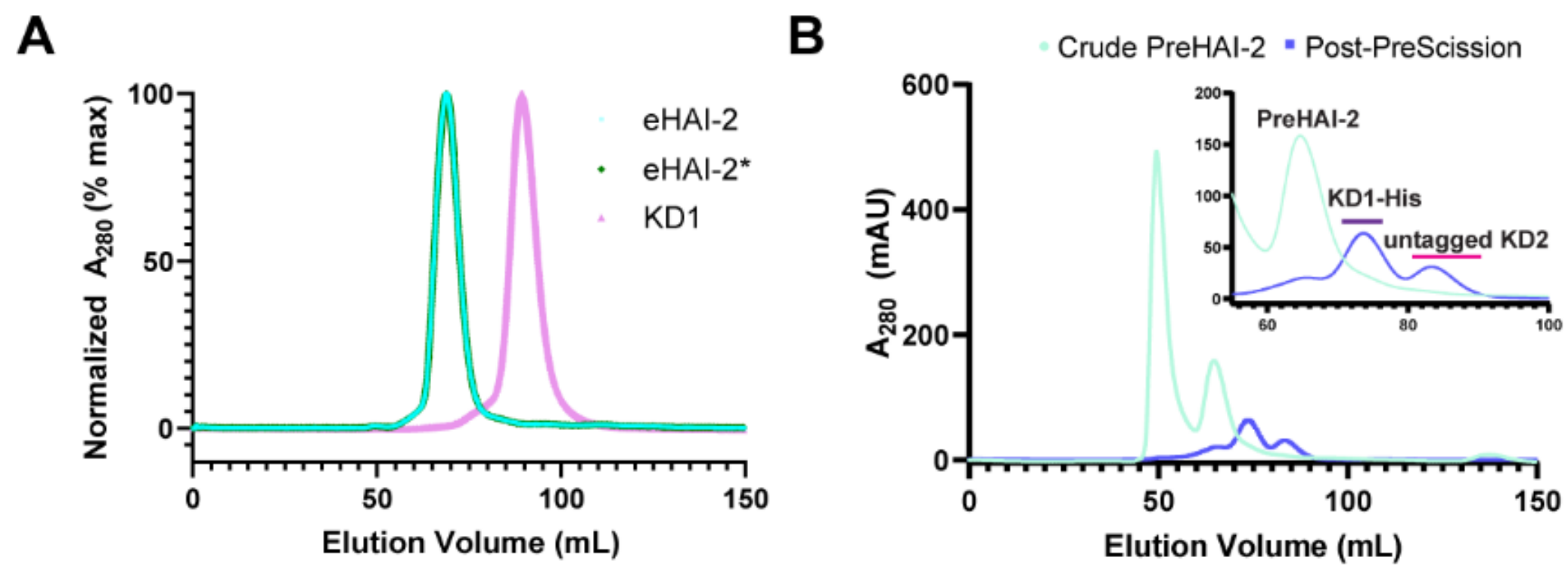

Fig. S1. Size-exclusion chromatography separation of HAI-2 and PreHAI-2 proteins. **A**, Size-exclusion chromatography chromatograms of the purified eHAI-2-His (teal), eHAI-2*-His (green) and KD1-His (pink) proteins. All proteins were loaded to a HiLoad 16/60 Superdex 75 gel filtration column. Protein content, monitored by A_{280}, was normalized to the maximum observed signal and plotted as a percentage. **B**, Size-exclusion chromatography profiles of crude PreHAI-2 (teal) overlaid with purified PreHAI-2 treated with PreScission Protease for 38 hours (blue) to facilitate untagged KD2 domain release. Bars indicate the fractions pooled for isolation of untagged KD2 (pink) and TEV-His-KD1 (purple).

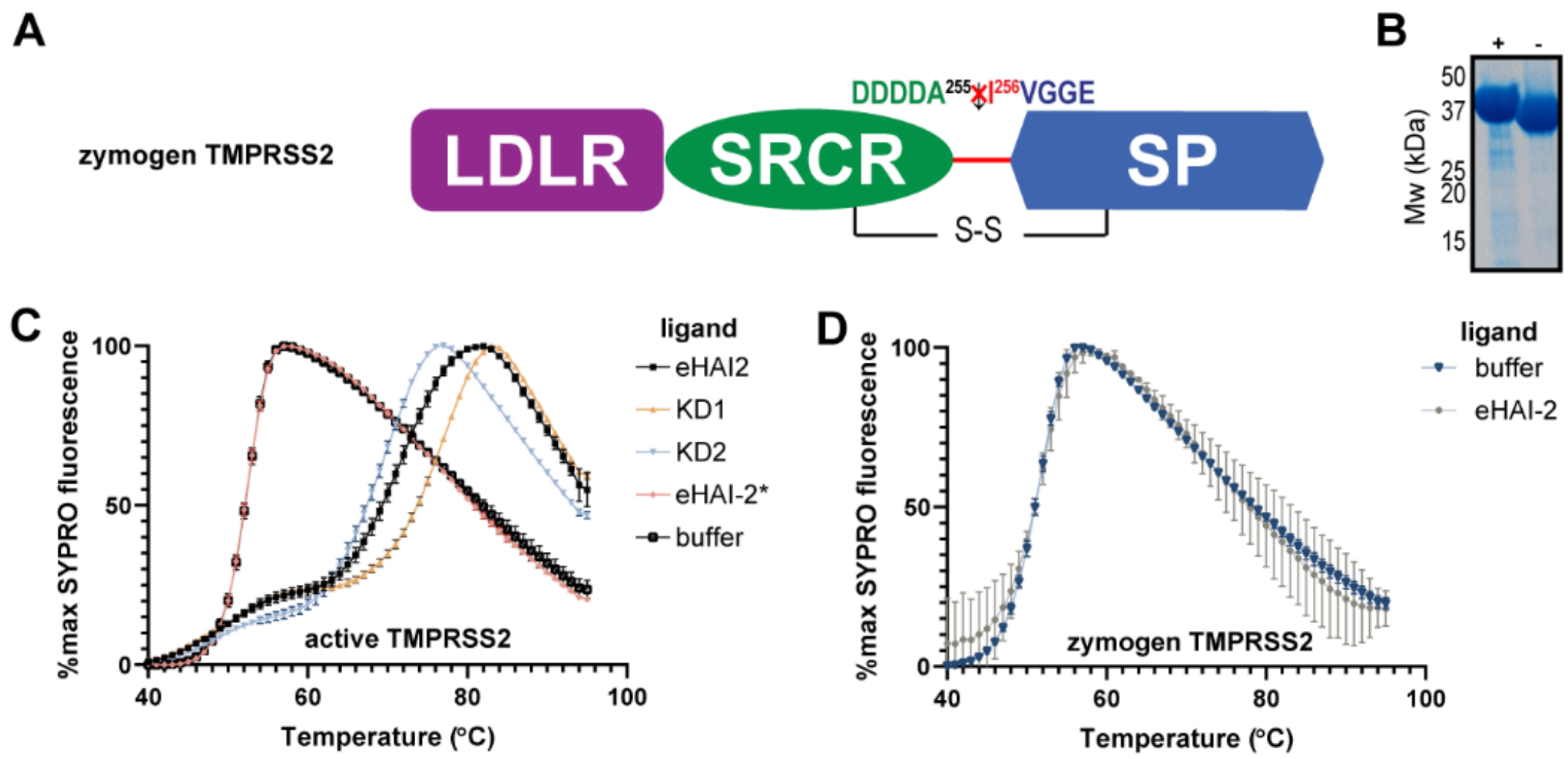

Fig. S2. Design of a zymogen-locked TMPRSS2 protein and DSF ligand binding assays with zymogen and active TMPRSS2. A, Schematic of the zymogen TMPRSS2 protein construct with a DDDDA255 amino acid substitution within the TMPRSS2 zymogen activation motif. **B**, SDS-PAGE analysis of the purified zymogen TMPRSS2 protein. SDS-PAGE samples were thermally denatured and reduced (5 mM 2-mercaptoethanol, 5 min 95 C; indicated with +) or were not thermally denatured and reduced (indicated with -) prior to gel separation. **C**, TMPRSS2 melt curves in the presence of the indicated HAI-2 protein or vector control (buffer), detected by fluorescence (excitation:emission of 465:580) with the fluorogenic SYPRO orange dye. Approximately 2 µg active TMPRSS2 and 5X SYPRO orange were used per well. **D**, Zymogen TMPRSS2 melt curves in the presence of eHAI-2 or vector control (buffer).

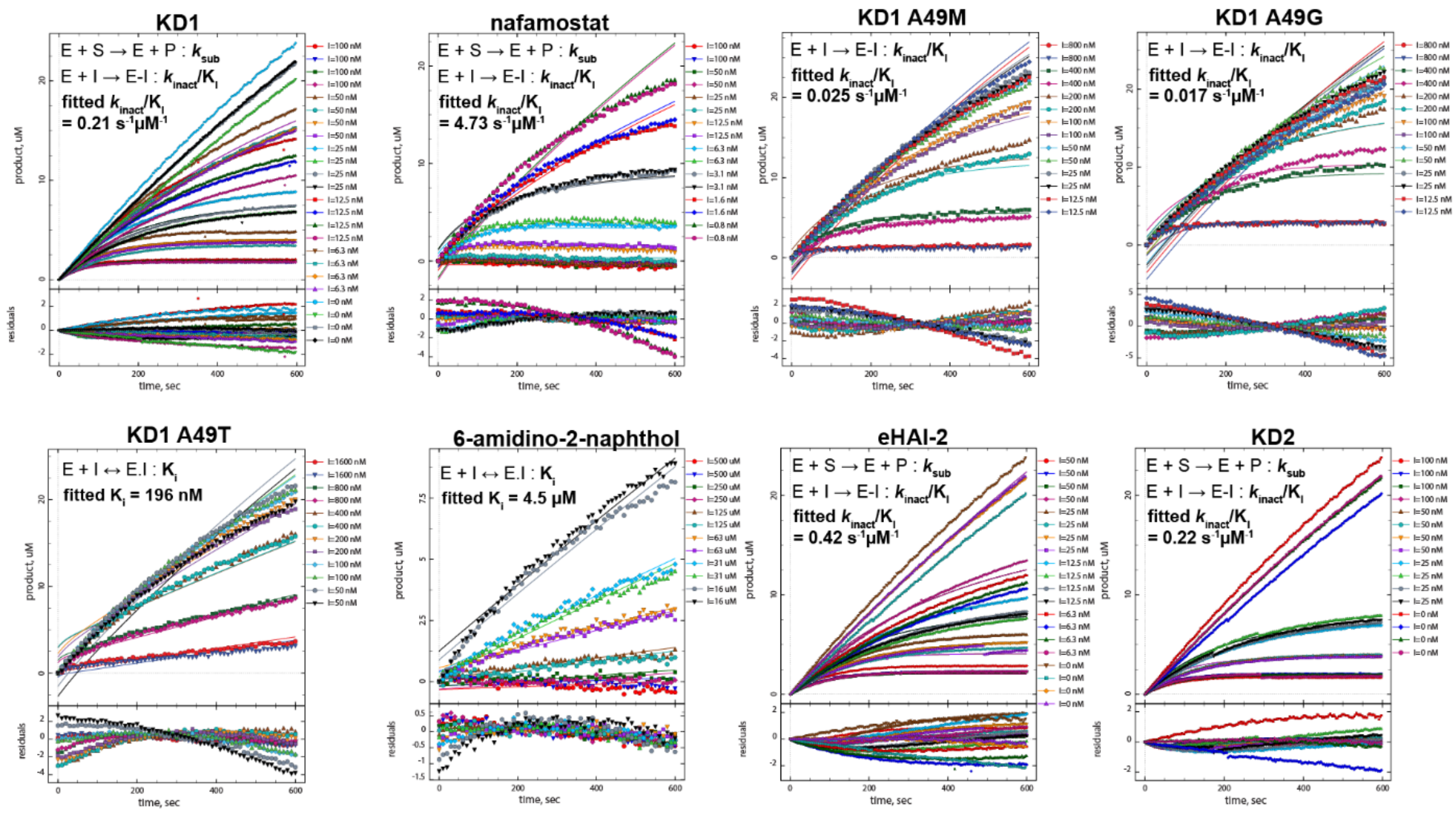

Fig. S3. TMPRSS2 inactivation kinetics with HAI-2 proteins and small molecule serine protease inhibitors. Kinetic model and curve-fitted data for TMPRSS2 incubated with the indicated concentrations of HAI-2 protein domains or small molecule inhibitors, measured through TMPRSS2 activity towards 100 µM fluorogenic Boc-QAR-AMC substrate. Model parameters for curve fitting are shown, with residual values from the data curve fitting shown below the plot.

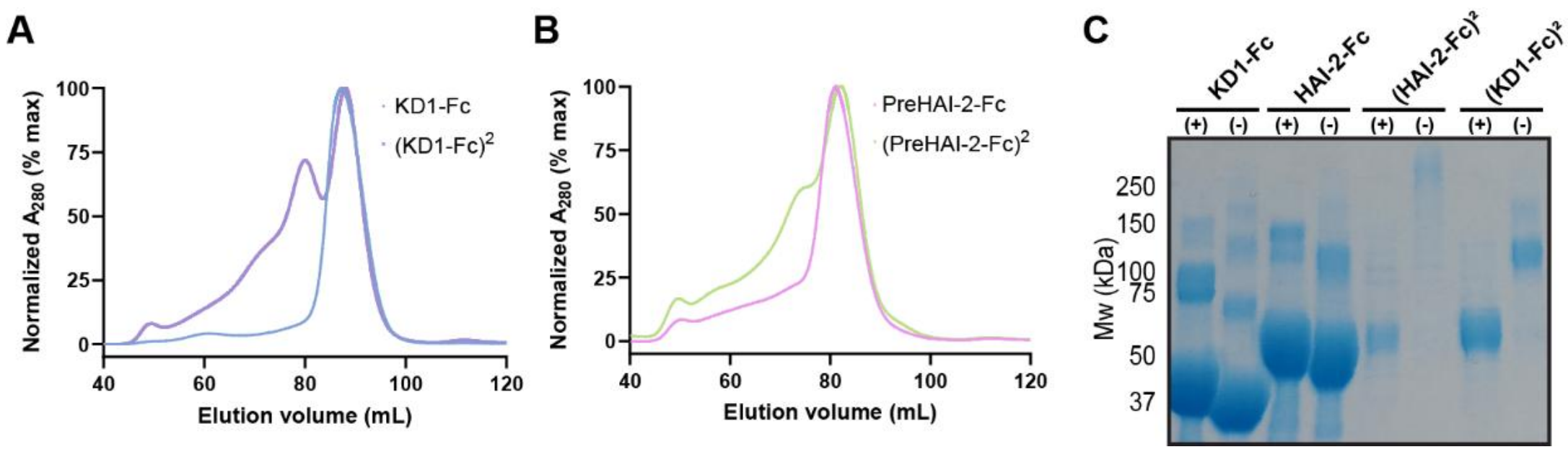

Fig. S4. Purification of Fc-tagged HAI-2 proteins secreted from Expi293F cells. **A**, Size-exclusion chromatography (SEC) purification of the KD1-Fc (teal) and (KD1-Fc)^2 (violet) proteins. Proteins were loaded to a HiLoad 16/60 Superdex 75 gel filtration column. Protein content, monitored by A_{280}, was normalized to the maximum observed signal and plotted as a percentage. **B**, SEC purification of the eHAI-2-Fc (pink) and (eHAI-Fc)^2 (lime) proteins. **C**, SDS-PAGE analysis of purified, Fc-tagged HAI-2 proteins. Prior to SDS-PAGE separation, samples were thermally denatured and reduced (+) or not thermally denatured and not reduced (-).

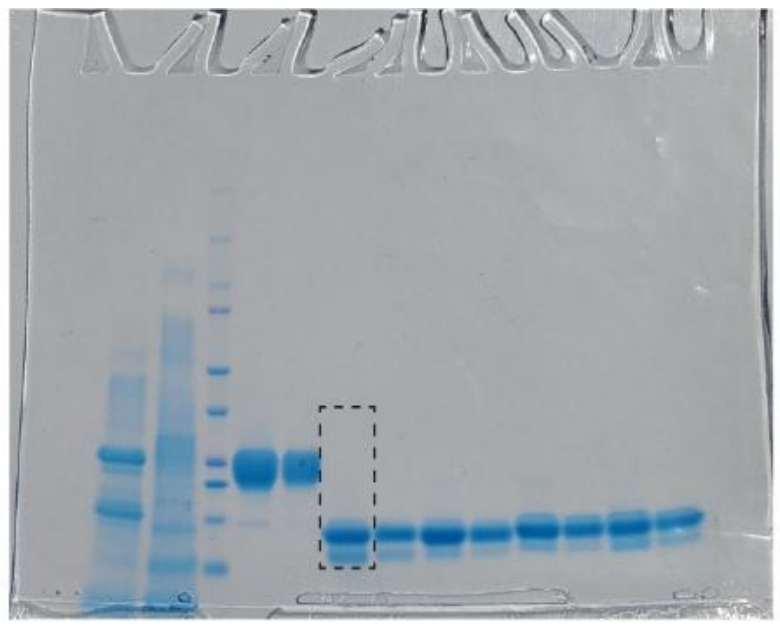

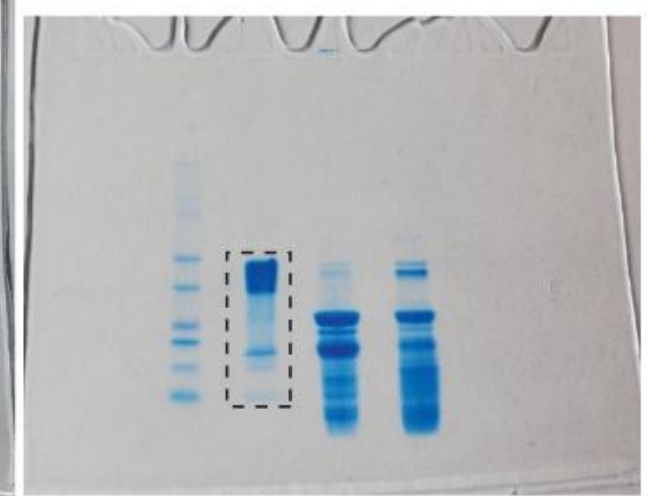

Fig. 5B

Fig. 3A

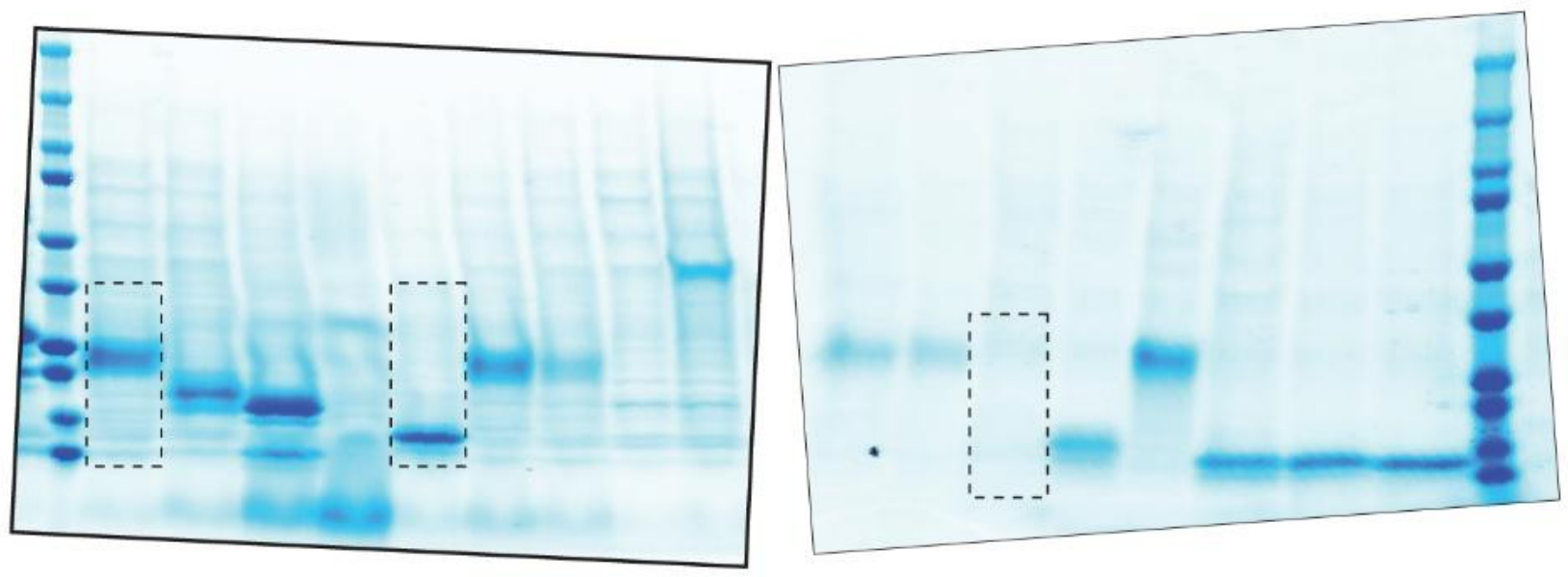

Fig. 7B

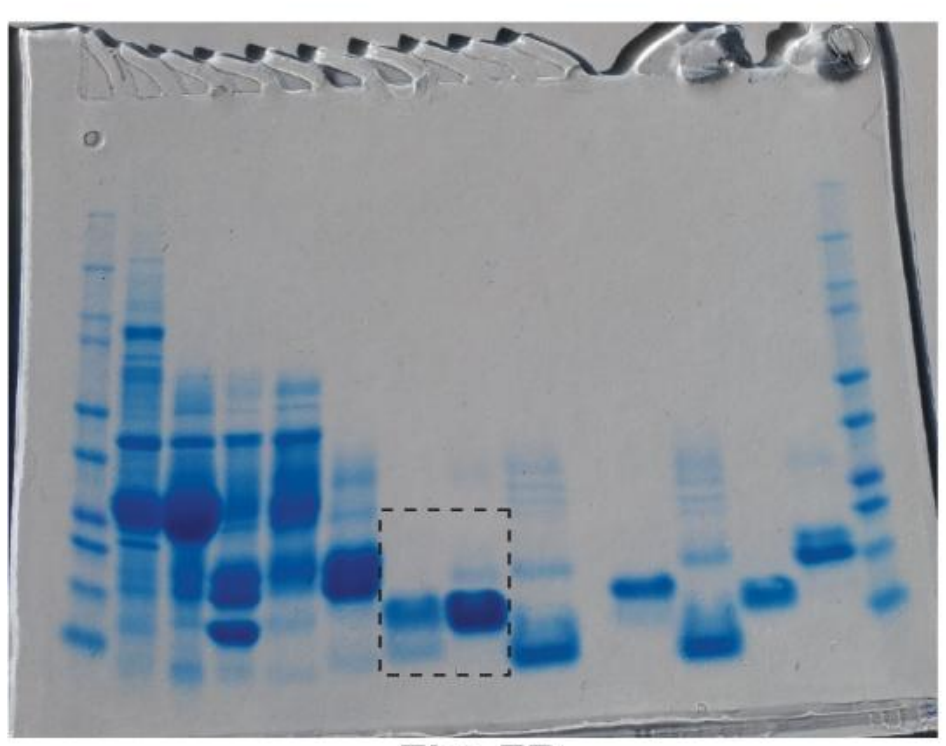

Fig. 7B

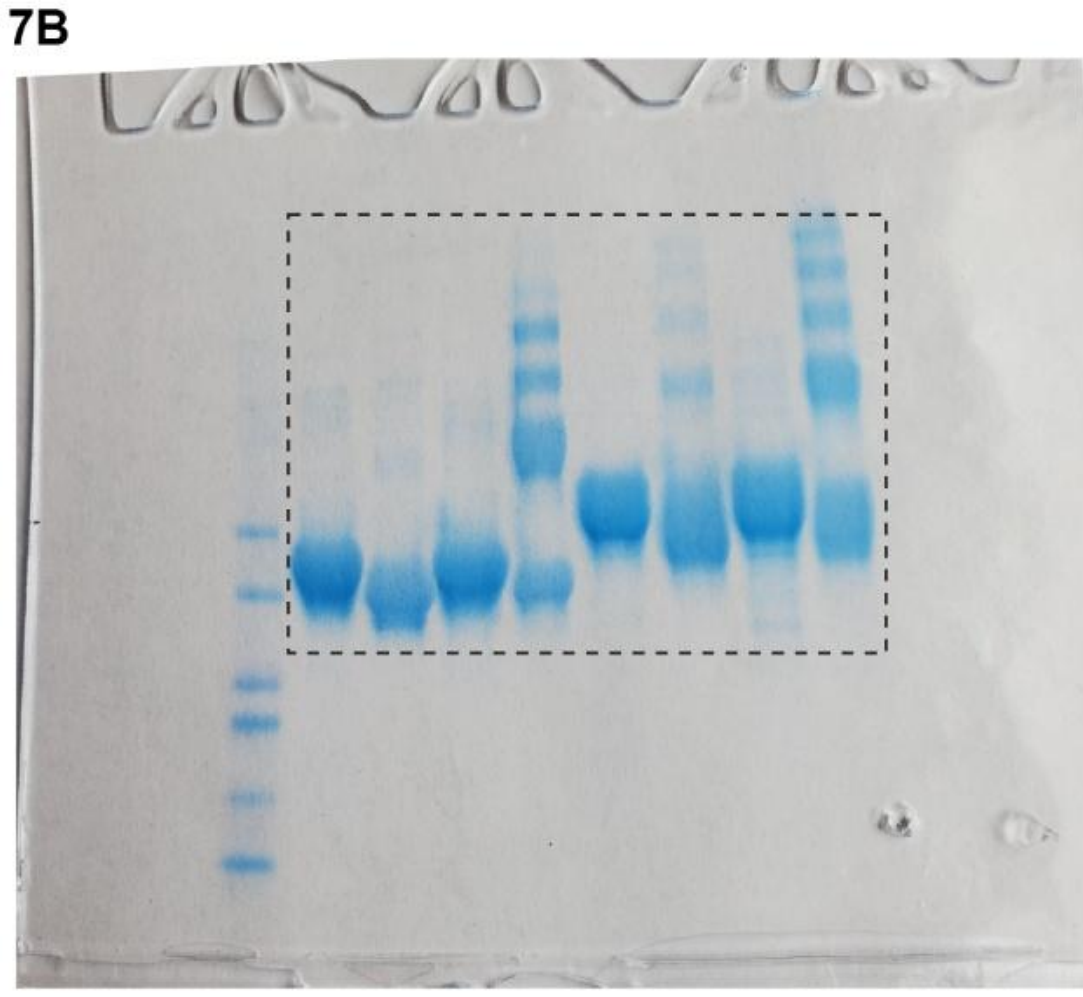

Fig. 8C

Fig. S5. Uncropped gel images

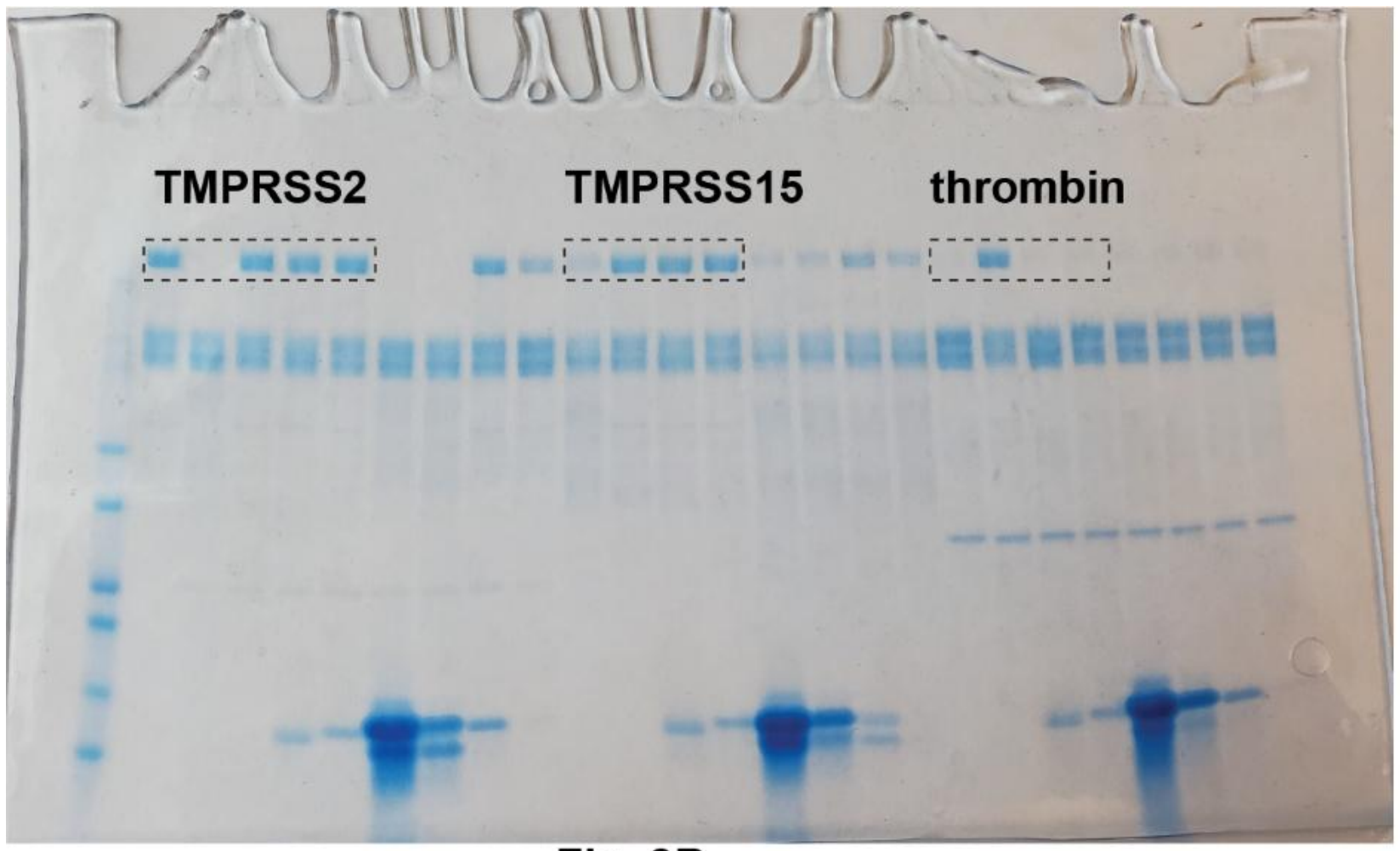

Fig. 9B

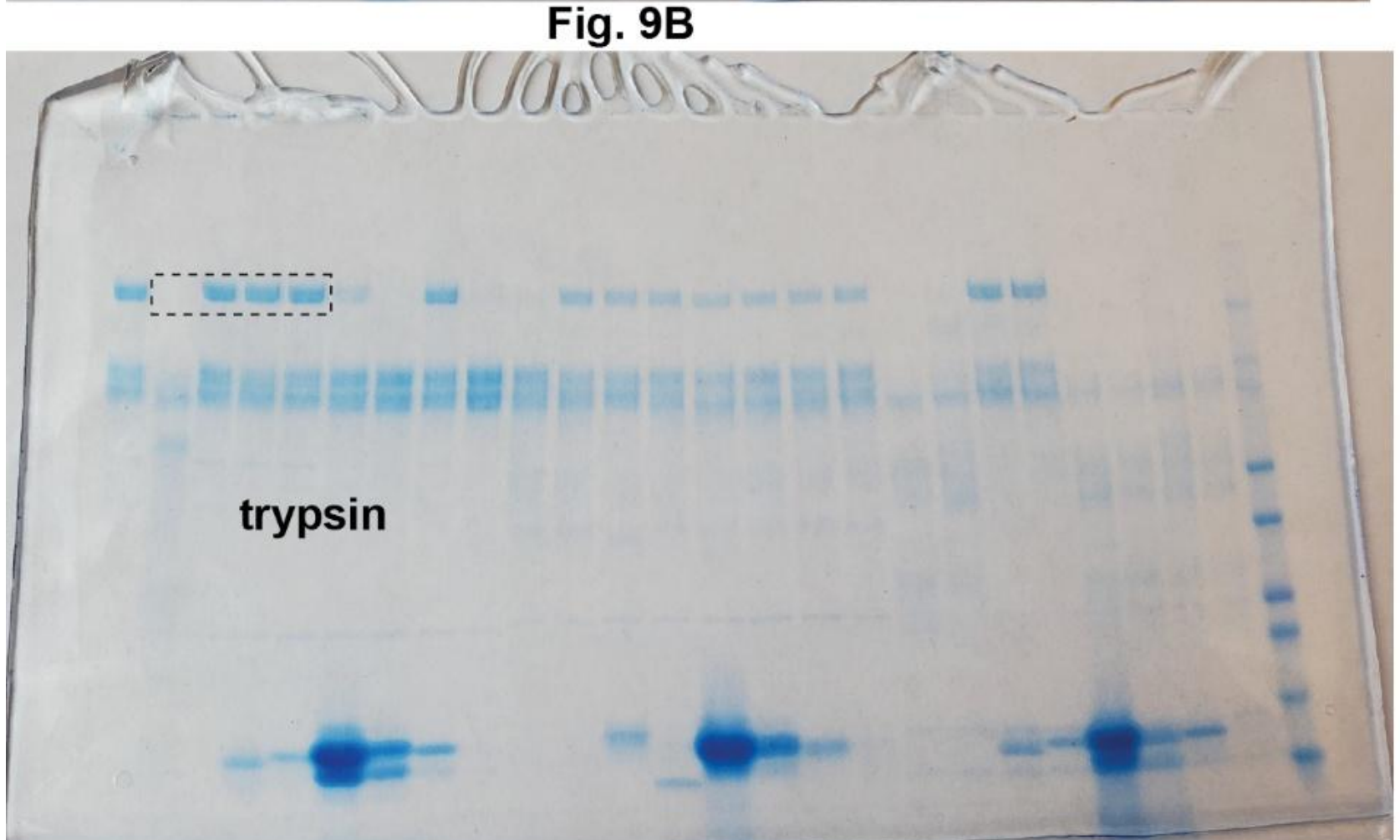

Fig. 9B

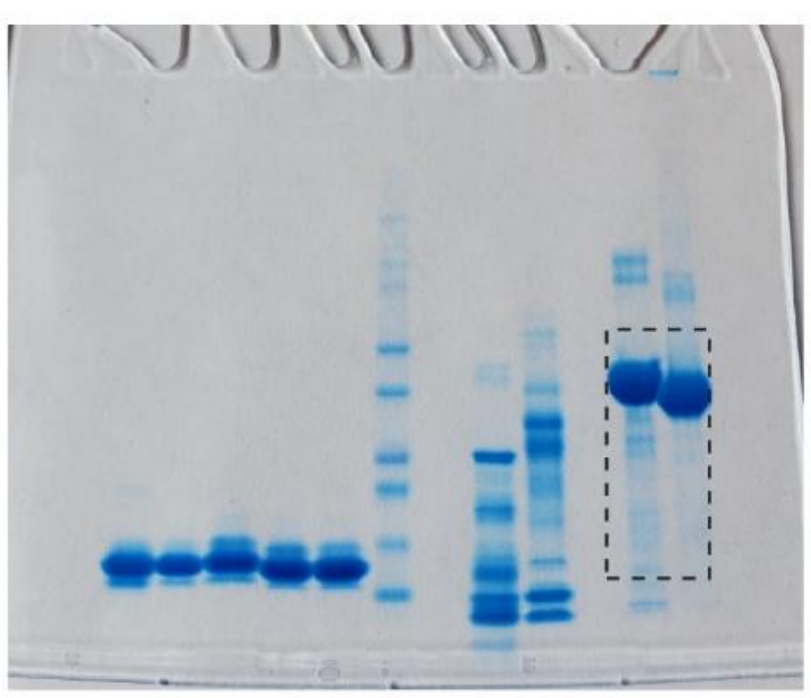

Fig. S2B

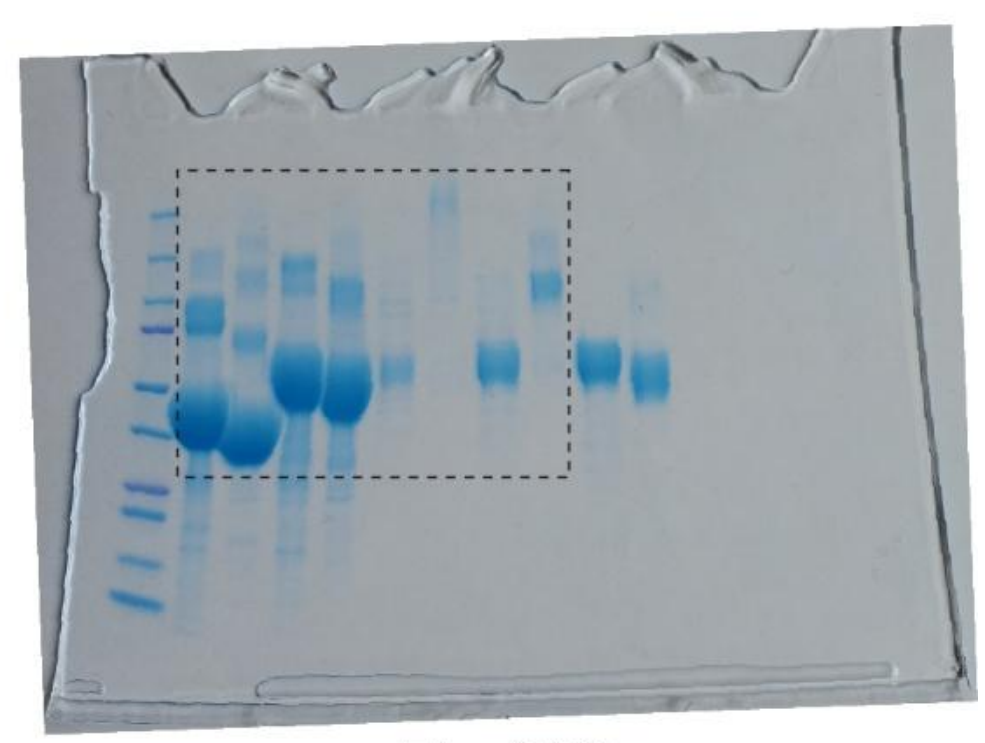

Fig. S4C

Fig. S6. Uncropped gel images

Table S1. Ligation independent cloning (LIC) mutagenesis primer sequences for recombinant HAI-2 and TMPRSS2 protein constructs. All constructs contain a N-terminal honeybee mellitin signal sequence and a C-terminal EFVEHHHHHHHHH affinity tag, unless otherwise indicated.

**Protein construct (residues)**		**Primer sequence (5' to 3')**
eHAI-2 (29-197)	Fwd	tacatctatgcggccgctgaccgagaacgcagcatcc
	Rev	ggtgctcgacgaattcctttgagccaaggggcagg
HAI-2 KD1 (29-91)	Fwd	tacatctatgcggccgctgaccgagaacgcagcatcc
	Rev	ggtgctcgacgaattcgacagtggcacatttcttgag
HAI-2 KD2 (130-186)	Fwd	tacatctatgcggccgctgaagaatactgcaccgccaac
	Rev	ggtgctcgacgaattcctggcggaagcagcggag
eHAI-2* (29-197)	Gene synthesized	
PreHAI-2†	Fwd (1;2)	ttgtatttccagggcgaccgagaacgcagcatcc; agcaggctcgaagttcttttccagggaccaagtgctcccagaaggcagg
	Rev (1;2)	acttggtccctggaaaagaacttcgagcctgctggtggccaggtcacc; caagcttcgtcatcactttgagccaaggggcagg
HAI-2 KD1 A49G (29-91)	Fwd	tacatctatgcggccgctgaccgagaacgcagcatcc
	Rev	ggtgctcgacgaattcgacagtggcacatttcttgag
HAI-2 KD1 A49S (29-91)	Fwd (1;2)	tacatctatgcggccgctgaccgagaacgcagcatcc; tgccggagctccatgcctaggtggtggtac
	Rev (1;2)	catggagctccggcatctgcccaccaccttcg; ggtgctcgacgaattcgacagtggcacatttcttgag
HAI-2 KD1 A49M (29-91)	Fwd	tacatctatgcggccgctgaccgagaacgcagcatcc
	Rev	ggtgctcgacgaattcgacagtggcacatttcttgag
HAI-2 KD1 A49T (29-91)	Fwd	tacatctatgcggccgctgaccgagaacgcagcatcc
	Rev	ggtgctcgacgaattcgacagtggcacatttcttgag
Zymogen TMPRSS2 (109-492)†	Fwd (1;2;3)	tacatctatgcggccgctatgagcggcctgaacg; attgaatggcatgaaatgggcagcaagtgctccaac; gacgacgcgatcgtgggcggcgagagcgcgctc
	Rev (1;2;3)	ttcatgccattcaattttctgcg; acgatcgcgtcgtcgtcgtctgagttcaagttga; ggtgctcgacgaattcgccgtctgccctcatttgtcg
